# Association of HARMS_2_-AF score with atrial tachyarrhythmia recurrence after radiofrequency catheter ablation: a retrospective observational study

**DOI:** 10.1186/s12872-025-05110-y

**Published:** 2025-08-28

**Authors:** Ying Zhu, Meiqi Miao, Zhaochen Xia, Shiyu Liu, Cheng Chang, Licheng Lu, Rong Qian, Jianfeng Qian, Haixiang Xu, Wen Pan, Jianhua Fan

**Affiliations:** Department of Cardiology, Kunshan Hospital of Traditional Chinese Medicine, Suzhou, Jiangsu China

**Keywords:** Atrial fibrillation, Radiofrequency catheter ablation, Recurrence, HARMS_2_-AF score

## Abstract

**Background and objective:**

Atrial fibrillation (AF) is the most common sustained arrhythmia, with high recurrence rates following radiofrequency catheter ablation (RFCA). Identifying predictors of atrial tachyarrhythmia (Ata) recurrence is crucial for risk stratification and personalized management. The HARMS_2_-AF score, a novel lifestyle-based risk score comprising hypertension, age, BMI ≥ 30 kg/m², male sex, sleep apnea, smoking, and alcohol consumption, has emerged as a potential predictor for Ata recurrence. This study aimed to evaluate the association between the HARMS_2_-AF score and Ata recurrence after RFCA.

**Methods:**

We conducted a retrospective analysis of 152 patients who underwent RFCA at Kunshan Hospital of Traditional Chinese Medicine between January 2021 and December 2022. Ata recurrence was defined as documented episodes of atrial flutter, atrial tachycardia, or AF lasting more than 30 s on ECG or 24-hour Holter monitorin during follow-up. Based on Ata recurrence, patients were classified into recurrence (*n* = 44) and non-recurrence (*n* = 108) groups. Clinical characteristics, CHA_2_DS_2_-VASc score, and HARMS_2_-AF score were compared between the two groups. Spearman’s rank correlation analysis was performed to assess the relationships between the HARMS_2_-AF score, CHA_2_DS_2_-VASc score, left atrial diameter (LAD), and Ata recurrence. Univariate and multivariate logistic regression analyses were performed to identify independent predictors of Ata recurrence.

**Results:**

Patients in the recurrence group exhibited a higher prevalence of persistent AF (*P* = 0.002), larger LAD (*P* < 0.001), and higher CHA_2_DS_2_-VASc (*P* < 0.001) and HARMS_2_-AF scores (*P* < 0.001) compared to the non-recurrence group. Spearman’s rank correlation analysis revealed significant positive correlations between the HARMS_2_-AF score (*r* = 0.626, *P* < 0.001), CHA_2_DS_2_-VASc score (*r* = 0.452, *P* < 0.001), and LAD (*r* = 0.405, *P* < 0.001) with Ata recurrence. Multivariate analysis revealed that LAD (OR = 1.280, 95% CI = 1.118–1.464), CHA_2_DS_2_-VASc (OR = 3.773, 95% CI = 1.897–7.503), and HARMS_2_-AF (OR = 3.106, 95% CI = 1.866–5.168) were independent predictors for Ata recurrence after RFCA. The HARMS_2_-AF score demonstrated high sensitivity (93.2%) and specificity (79.6%) for predicting Ata recurrence. The area under the receiver operating characteristic (ROC) curve (AUC) was 0.895 for HARMS_2_-AF, 0.777 for CHA_2_DS_2_-VASc, and 0.757 for LAD, with HARMS_2_-AF showing superior predictive accuracy (*P* = 0.008 vs. CHA_2_DS_2_-VASc, *P* = 0.007 vs. LAD).

**Conclusion:**

The HARMS_2_-AF score is significantly associated with Ata recurrence after RFCA and provides a valuable tool for risk prediction. A cut-off value of 7.5 for the HARMS_2_-AF score demonstrates high sensitivity and specificity for predicting Ata recurrence, offering superior prognostic value compared to traditional risk factors. Additionally, Spearman’s rank correlation analysis confirms the strong relationship between lifestyle-related factors (as captured by the HARMS_2_-AF score) and Ata recurrence, further supporting the clinical relevance of this score.

## Introduction

Atrial fibrillation (AF) is a predominant cardiac arrhythmia that poses significant risks for various cardiovascular complications, including stroke and heart failure [1], particularly among elderly populations. This condition has a substantial impact on patients’ quality of life and contributes heavily to healthcare expenditures due to its associated morbidity and mortality. Therefore, early implementation of effective treatment for AF patients is of great significance, as it is key to controlling disease progression and improving prognosis [2].

Despite advancements in therapeutic approaches, including anticoagulation therapy and radiofrequency catheter ablation(RFCA), AF remains challenging to treat due to its high recurrence rates [3]. The recurrence of atrial tachyarrhythmia (Ata) after RFCA can lead to repeated hospitalizations and the need for additional interventions, thereby complicating patient management and affecting quality of life. Identifying patients at high risk for Ata recurrence after RFCA is crucial for optimizing treatment and improving long-term outcomes. Traditional risk scores, such as the CHA_2_DS_2_-VASc score, have been widely used to assess the risk of thromboembolic events in AF patients. However, their ability to predict Ata recurrence following RFCA remains limited, as they primarily focus on thromboembolic risks and fail to account for lifestyle and modifiable risk factors that are crucial in Ata recurrence.

There have been reports in the literature that improvements in lifestyle after AF ablation, such as strict abstinence from alcohol, enhancing cardiorespiratory fitness, controlling body mass index and blood pressure, and improving obstructive sleep apnea, can to some extent reduce the recurrence rate of AF [4, 5]. The HARMS_2_-AF score, a novel lifestyle risk score, may help identify individuals at risk of AF [6]. The HARMS_2_-AF score comprises seven variables: hypertension (4 points), age (1–2 points depending on age group), body mass index (BMI) ≥ 30 kg/m² (1 point), male sex (2 points), sleep apnea (2 points), smoking (1 point), and alcohol consumption (1–2 points depending on weekly intake), yielding a total possible score of 14. The score has shown promising results in cohort studies, such as those utilizing data from large populations like the UK Biobank, where it was validated as an effective tool for stratifying AF risk based on lifestyle factors. However, there has been limited research specifically focusing on its role in predicting Ata recurrence after RFCA. Our study seeks to address this gap by exploring the association between the HARMS_2_-AF score and Ata recurrence post-RFCA, contributing to the growing body of evidence supporting its clinical utility.

## Materials and methods

### Study population

The diagnosis of AF was based on electrocardiogram (ECG) or 24-hour Holter monitoring. The definition of paroxysmal atrial fibrillation(PAF)and persistent atrial fibrillation (PsAF) is consistent with current guidelines [7]. Inclusion criteria: Patients aged between 18 and 80 years who were diagnosed with AF and underwent RFCA at Kunshan Hospital of Traditional Chinese Medicine between January 2021 and December 2022. The following patients were excluded from the final analysis: those with valvular AF, preoperative left atrial thrombus, contraindications to anticoagulation, comorbid dilated cardiomyopathy, ischemic cardiomyopathy, or hypertrophic cardiomyopathy, failure to restore sinus rhythm after RFCA, prior history of RFCA, incomplete clinical data or follow-up, and those with severe hepatic or renal insufficiency, thyroid dysfunction, severe chronic respiratory conditions—such as chronic obstructive pulmonary disease (COPD), interstitial lung disease, or pulmonary fibrosis, or a history of malignancy. Additionally, patients with a left atrial diameter greater than 55 mm were excluded from the study. The primary endpoint was the occurrence of Ata recurrence, defined as documented episodes of atrial flutter, atrial tachycardia, or AF lasting more than 30 s on ECG or 24-hour Holter monitoring during follow-up at 3, 6, and 12 months post-RFCA [8]. Patients were classified into a recurrence group or a non-recurrence group based on the presence or absence of Ata recurrence during follow-up.

The study was approved by the Ethics Committee of Kunshan Hospital of Traditional Chinese Medicine. Since this was a retrospective study and no new treatment interventions were performed on patients, the requirement for obtaining informed consent was waived after review by the ethics committee. All methods were carried out in accordance with relevant guidelines and regulations.

### Ablation procedure

RFCA procedures were performed by two expert electrophysiologists under conscious sedation. All patients underwent transesophageal echocardiography (TEE) one day prior to the procedure to rule out left atrial thrombus. In cases where TEE was not tolerated, intracardiac echocardiography (ICE) was performed intraoperatively to exclude left atrial thrombus before proceeding with ablation. Following transseptal access, an electroanatomical map of the left atrium was created using a 3D navigation system (CARTO3, Biosense Webster, Diamond Bar, CA, USA). Pulmonary vein isolation (PVI) was achieved using a 3.5 mm irrigated-tip RFCA catheter (Thermocool Smarttouch SF or Thermocool Smarttouch; Biosense Webster), and the procedural endpoint was the absence of any pulmonary vein (PV) potential recorded by a spiral mapping catheter (Pentary, Biosense Webster) placed along the ostium of the respective PV following a waiting period of 30 min. In cases where the distance between the top of the left and right pulmonary veins was short after wide area circumferential PVI, we routinely performed a left atrial roof line ablation to ensure adequate isolation and prevent potential re-entrant circuits. Additional linear lesions were applied in cases where PVI alone failed to restore sinus rhythm (SR). The selection of ablation lines—such as the roof line, bottom line, or mitral isthmus line—was based on individualized electrophysiological findings. If SR was achieved during roof line ablation, bottom line ablation was omitted. When SR could not be restored after linear ablation, direct-current cardioversion was performed. Cavotricuspid or mitral isthmus ablation was performed if atrial flutter or atrial tachycardia was documented preoperatively or identified intra-procedurally. Acute procedural success was defined as complete electrical isolation of all PVs and bidirectional block across any additional linear lesions.

### HARMS_2_-AF score and CHA_2_DS_2_-VASc score

The HARMS_2_-AF score includes several lifestyle and comorbidity risk factors: hypertension (4 points), age (60–64 years = 1 point, ≥ 65 years = 2 points), BMI ≥ 30 kg/m² (1 point), male sex (2 points), sleep apnea (2 points), smoking (1 point), and alcohol consumption (7–14 standard drinks/week = 1 point, ≥ 15 standard drinks/week = 2 points), with a total possible score of 14 points(1). The CHA_2_DS_2_-VASc score includes the following parameters: congestive heart failure, hypertension, age ≥ 75 years (2 points), type 2 diabetes, previous stroke/transient ischemic attack/thromboembolism (2 points), vascular disease, age 65–75 years, and female sex. All parameters for both scores were retrospectively collected from patients’ electronic medical records and outpatient records at the time of study inclusion, prior to their radiofrequency catheter ablation procedure, ensuring consistency and accuracy of the data. If any data for these scores were missing, the missing values were handled using mean imputation for continuous variables and mode imputation for categorical variables. If more than 30% of the data for a given patient were missing, the patient was excluded from the analysis.

### Follow-up

Patients had regular follow-ups at 1, 3, 6, and 12 months post-RFCA or whenever symptoms occurred, with ECG performed at each visit and 24-hour ECG monitoring at 3, 6, and 12 months. If clinical data were insufficient, follow-up was supplemented with telephonic interviews.

Oral anticoagulation was resumed 6 h post-ablation and maintained for 3 months. After this period, the decision to continue or discontinue anticoagulation was based on the CHA_2_DS_2_-VASc score: Patients with a score of ≥ 2 were advised to continue anticoagulation, while those with a score of < 2 were advised to discontinue anticoagulation. Class I/III antiarrhythmic drugs were prescribed for the first 3 months post-procedure. The decision to continue these medications was made based on follow-up assessments at 3 months, where patients showing Ata or significant arrhythmias on ECG or Holter monitoring continued the medications.

### Statistical analysis

Statistical analyses were conducted using SPSS 20.0 software. Continuous variables were expressed as mean ± standard deviation, and comparisons between groups were performed using t-tests for normally distributed data or Mann-Whitney U tests for non-normally distributed data. Shapiro-Wilk tests were used to assess normality. Categorical data were presented as counts and proportions, and were compared using the χ2 test or Fisher’s exact test when any cell had an expected frequency of less than 5. Spearman’s rank correlation coefficient was used to assess the relationship between the HARMS_2_-AF score, CHA_2_DS_2_-VASc score, LAD, and Ata recurrence. Multivariate logistic regression analysis was employed to identify independent predictors of Ata recurrence after RFCA, with the HARMS_2_-AF score, left atrial diameter (LAD), and CHA_2_DS_2_-VASc score included as key variables in the model. Receiver operating characteristic (ROC) curve analysis was further utilized to assess the predictive accuracy of the HARMS_2_-AF score relative to other variables. The Youden index (calculated as sensitivity + specificity − 1) was used to identify the cut-off point that maximized both sensitivity and specificity for predicting Ata recurrence at 12 months post-RFCA. Statistical significance was set at *P* < 0.05 for all analyses.

## Results

### Clinical characteristics of patients with and without the recurrence of AF

**Fig. 1:**
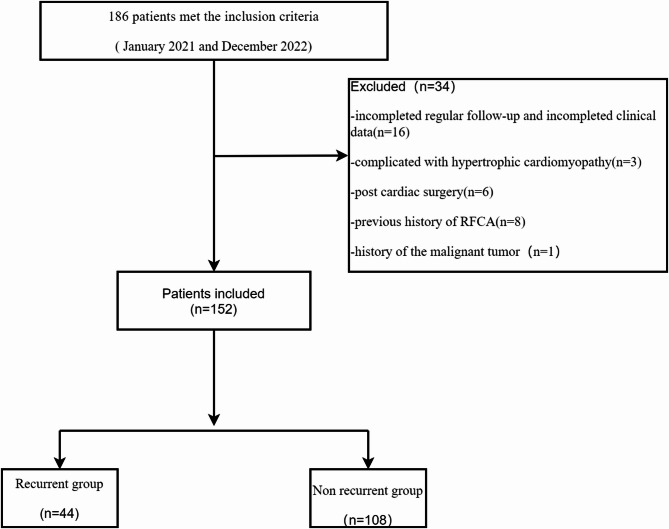
Flow chart detailing the inclusion and exclusion criteria

All 152 patients achieved sinus rhythm during the procedure and completed a 12-month follow-up (Fig. 1). During the study period, 44 patients (28.95%) experienced a documented recurrence of arrhythmia, while 108 patients (71.05%) remained arrhythmia-free. Table 1 presents the baseline characteristics of patients based on Ata recurrence status.

**Table 1:** Clinical characteristics of patients

Item	Recurrent group (*n* = 44)	Non recurrent group (*n* = 108)		χ^2^/t	*p*
Male[(n)%]	29(65.9%)		65(69.1%)	0.434	0.510
Age(years)	67.27 ± 10.78		65.57 ± 9.90	−0.935	0.351
PAF[(n)%]	20(45.5%)		78(72.2%)	9.780	0.002
Duration of AF(months)	53.32 ± 69.99		43.33 ± 80.07	−0.722	0.471
NYHA[(n)%]				1.455	0.483
I	21(47.7%)		58(53.7%)		
II	16(36.4%)		40(37.0%)		
III	7(15.9%)		10(9.3%)		
Hypertension[n(%)]	31(70.5%)		73(67.6%)	0.119	0.731
Diabetes[n(%)]	6(13.6%)		26(24.1%)	2.049	0.152
History of cerebrovascular disease[n(%)]	6(13.6%)		14(9.3%)	0.636	0.425
Peripheral vascular disease[n(%)]	2(4.5%)		3(2.8%)	0.289	0.451
Smoking[n(%)]	23(52.3%)		43(39.8%)	1.975	0.160
Drinking[n(%)]	14(31.8%)		22(20.4%)	2.267	0.132
BMI(kg/m^2^)	24.58 ± 3.28		24.52 ± 3.02	0.247	0.916
Sleep apnoea[n(%)]	4(9.1%)		2(1.9%)	3.891	0.059
History of thyroid disease[n(%)]	1(2.3%)		1(0.9%)	0.396	0.497
LAD (mm)	43.23 ± 5.63		37.95 ± 4.99	−5.695	<0.001
LVEDD (mm)	49.69 ± 4.62		47.94 ± 4.79	−2.058	0.041
LVESD(mm)	33.24 ± 5.80		32.47 ± 5.86	−0.737	0.462
LVEF(%)	56.26 ± 6.90		59.76 ± 7.07	2.787	0.006
Ablation strategy[n(%)]					
roof line ablation	23(52.3%)		45(41.7%)	1.422	0.233
bottom line ablation	22(50.0%)		41(38.0%)	1.867	0.172
mitral isthmus ablation	18(40.9%)		23(21.3%)	6.105	0.013
tricuspid isthmus ablation	3(6.8%)		8(7.4%)	0.016	0.899
electrical cardioversion	17(38.6%)		21(19.4%)	6.141	0.013
Postoperative medications[n(%)]					
amiodarone	15(34.1%)		35(32.4%)	0.040	0.841
propafenone	8(18.2%)		10(9.3%)	2.384	0.123
rivaroxaban	32(72.7%)		78(72.2%)	0.004	0.950
dabigatran	12(27.3%)		30(27.8%)	0.004	0.950
CHA_2_DS_2−_-VASc	4.64 ± 0.81		3.55 ± 1.10	−5.958	<0.001
HARMS_2_-AF	8.98 ± 1.32		5.81 ± 2.01	−9.638	<0.001

Values are presented as mean ± SD or n (%), *PAF* paroxysmal atrial fibrillation; NYHA: New York Heart Association; BMI: Body Mass Index, *NT-proBNP* N-terminal pro-B type natriuretic peptide, *LAD* left atrial diameter, *LVEDD* left ventricular end diastolic diameter, *LVESD* left ventricular end systolic diameter, *LVEF* left ventricular ejection fraction,*χ*^*2*^*/t*” represents either the Chi-square (*χ²*) statistic for categorical variables or the t-statistic for continuous variables

In the recurrence group, 34.1% of patients were prescribed amiodarone, 18.2% received propafenone. In contrast, the non-recurrence group had 32.4% amiodarone use and 9.3% propafenone use. The impact of these therapies on Ata recurrence was analyzed, and no statistically significant difference was found between the two groups in terms of recurrence rates associated with specific medications (*P* = 0.841 for amiodarone and *P* = 0.123 for propafenone).

Among the 44 patients in the recurrence group, 12 (27.3%) underwent a repeat ablation procedure due to sustained or recurrent AF. During these follow-up procedures, 6 patients showed recovery of PV potentials, 3 patients had atrial tachycardia dependent on the mitral isthmus, 1 patient had tachycardia originating from the left atrial roof, and 2 patients had AF originating from the superior vena cava. All of these patients underwent successful additional ablation procedures.

### Univariate and multivariate analysis of recurrence

In the univariate analysis, the recurrence group had a lower proportion of patients with PAF (*P* = 0.002), a larger LAD (*P* = 0.005), larger left ventricular end-diastolic diameter (LVEDD) (*P* = 0.041), lower left ventricular ejection fraction (LVEF) (*P* = 0.006), higher frequency of mitral isthmus ablation (*P* = 0.013), more cases of electrical cardioversion (*P* = 0.013), higher CHA_2_DS_2_-VASc scores (*P* = 0.012) and higher HARMS_2_AF scores (*P* = 0.015) (Table 1).

In the multivariate logistic regression analysis, LAD (OR = 1.280, 95% CI = 1.118–1.464, *P* < 0.001), CHA_2_DS_2_-VASc (OR = 3.773, 95% CI = 1.897–7.503, *P* < 0.001), and HARMS_2_-AF (OR = 3.106, 95% CI = 1.866–5.168, *P* < 0.001) were identified as independent predictors of Ata recurrence post-RFCA(Table 2).

**Table 2:** Multivariate analysis of recurrence of Ata after RFCA

Variable	B	SE	Wald χ^2^	OR	95%CI	*p*
PAF	−0.136	0.813	0.028	0.873	0.177–4.299	0.868
LAD	0.247	0.069	12.831	1.280	1.118–1.464	<0.001
LVEDD	−0.081	0.073	1.246	0.922	0.799–1.064	0.264
LVEF	0.036	0.039	0.843	1.037	0.960–1.120	0.359
mitral isthmus ablation	0.437	0.744	0.345	1.549	0.360–6.658	0.557
electrical cardioversion	0.677	0.850	0.634	1.967	0.372–10.408	0.426
CHA_2_DS_2_-VASc	1.328	0.351	14.334	3.773	1.897–7.503	<0.001
HARMS_2_-AF	1.133	0.260	19.013	3.106	1.866–5.168	<0.001

*PAF* paroxysmal atrial fibrillation, *LAD* left atrial diameter, *LVEDD* left ventricular end diastolic diameter, *LVEF* left ventricular ejection fraction, *B *regression coefficients; SE: standard errors,χ² Chi-square, *OR* odds ratio, *CI* confidence interval, *Ata* Atrial Tachyarrhythmia

The regression coefficient (*B*) represents the change in the log-odds of recurrence for each unit increase in the predictor variable. The standard error (*SE*) is the measure of the variability of the regression coefficient. The Wald chi-square (*χ*^*2*^) statistic tests the significance of each predictor, with a *p*-value indicating whether the predictor significantly contributes to AF recurrence. Odds ratios (*OR*) are reported along with their 95% confidence intervals (*CI*), showing the strength of association between each variable and Ata recurrence

### Predictive value of HARMS_2_-AF for Ata recurrence after RFCA

**Fig. 2:**
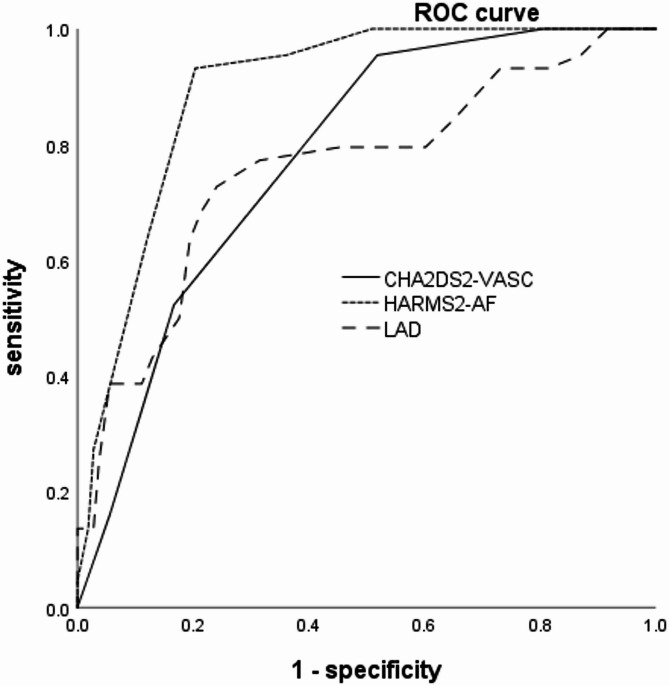
ROC curves for the prediction of Ata recurrence after RFCA

The area under the ROC curve (AUC) for predicting Ata recurrence post-RFCA was 0.895 for HARMS_2_-AF (95% CI = 0.846–0.944), 0.777 for CHA_2_DS_2_-VASc (95% CI = 0.703–0.851), and 0.757 for LAD (95% CI = 0.667–0.847). The optimal cut-off for HARMS_2_-AF was 7.5 (sensitivity = 93.2%, specificity = 79.6%), showing superior predictive value compared to CHA_2_DS_2_-VASc (AUC = 0.895 vs. 0.777, *P* = 0.008) and LAD (AUC = 0.895 vs. 0.757, *P* = 0.007). See Table 3; Fig. 2.

**Table 3:** ROC curves for LAD, CHA_2_DS_2_-VASc and HARMS_2_-AF in predicting Ata recurrence after RFCA

Variable	AUC	SE	95%CI	Cut-off value	sensitivity	specificity	*P*
LAD	0.757	0.046	0.667–0.847	40.5(mm)	0.727	0.759	0.007 ^#^
CHA_2_DS_2_-VASc	0.777	0.038	0.703–0.851	3.5	0.955	0.481	0.008 ^*^
HARMS_2_-AF	0.895	0.025	0.846–0.944	7.5	0.932	0.796	

*LAD* left atrial diameter, *Ata* Atrial Tachyarrhythmia

^*^indicates HARMS_2_-AF compare to CHA_2_DS_2_-VASc

^#^indicates HARMS_2_-AF compare to LAD

Receiver operating characteristic (*ROC*) curve analysis for left atrial diameter (*LAD*), CHA_2_DS_2_-VASc score, and HARMS_2_-AF score in predicting AF recurrence after RFCA. The area under the curve (*AUC*) represents the overall accuracy of each predictor in distinguishing between recurrence and non-recurrence groups, with higher AUC values indicating better predictive performance. The standard error (*SE*) measures the variability of the AUC estimate. The 95% confidence interval (*95% CI*) indicates the range within which the true AUC is expected to lie with 95% confidence

### Correlation between HARMS_2_-AF score, CHA_2_DS_2_-VASc score, LAD, and Ata recurrence

Spearman’s rank correlation analysis was performed to evaluate the relationships between the HARMS_2_-AF score, CHA_2_DS_2_-VASc score, LAD, and Ata recurrence. A strong positive correlation was found between the HARMS_2_-AF score and Ata recurrence (Spearman’s correlation coefficient = 0.626, *P* < 0.001), suggesting that higher scores are significantly associated with an increased risk of Ata recurrence. A moderate positive correlation was observed between the CHA_2_DS_2_-VASc score and Ata recurrence (Spearman’s correlation coefficient = 0.452, *P* < 0.001), indicating that patients with higher CHA_2_DS_2_-VASc scores are more likely to experience Ata recurrence. A weaker, but still statistically significant, positive correlation was found between LAD and Ata recurrence (Spearman’s correlation coefficient = 0.405, *P* < 0.001), suggesting that a larger LAD is associated with a higher risk of recurrence. The results are presented in Table 4.

**Table 4:** Correlation between HARMS_2_-AF, CHA_2_DS_2_-VASc, and LAD with Ata recurrence

Parameter	Correlation Coefficient	*P*
HARMS_2_-AF	0.626	< 0.001
CHA_2_DS_2_-VASc	0.452	< 0.001
LAD	0.405	< 0.001

*LAD* left atrial diameter, *Ata* Atrial Tachyarrhythmia

## Discussion

In this retrospective observational study, we demonstrated that the HARMS_2_-AF score is significantly associated with Ata recurrence following RFCA in patients with AF. Multivariate logistic regression analysis confirmed the HARMS_2_-AF score as an independent predictor of Ata recurrence, with an OR of 3.106 (95% CI: 1.866–5.168; *P* < 0.001). When compared to conventional risk factors such as LAD and the CHA_2_DS_2_-VASc score, the HARMS_2_-AF score exhibited superior predictive performance. The area under the ROC curve was 0.895, and a cut-off value of 7.5 yielded high sensitivity (93.2%) and specificity (79.6%). To further investigate the relationship between these variables, we performed Spearman’s rank correlation analysis.The correlation analysis revealed that the HARMS_2_-AF score had the strongest association with Ata recurrence (Spearman’s correlation coefficient = 0.626, *P* < 0.001), indicating that lifestyle factors like hypertension, BMI, and sleep apnea are significantly linked to recurrence risk. In comparison, the CHA_2_DS_2_-VASc score showed a moderate correlation (Spearman’s correlation coefficient = 0.452, *P* < 0.001), suggesting some predictive value for recurrence, though it was less predictive than the HARMS_2_-AF score. A weaker but still significant correlation was observed between left atrial diameter (LAD) and Ata recurrence (Spearman’s correlation coefficient = 0.405, *P* < 0.001), highlighting the role of structural heart changes in recurrence risk. Overall, these results demonstrate that the HARMS_2_-AF score, by incorporating lifestyle factors, offers superior predictive value for recurrence after RFCA.

Our results are consistent with those of Ko et al., who validated the HARMS_2_-AF score in a Japanese population using a large-scale epidemiological dataset [9]. Ko et al. found that the HARMS_2_-AF score showed good performance in predicting AF, with a Harrell’s C-index of 0.703, which was comparable to the C_2_HEST score developed specifically for Asian populations (C-index = 0.717). This indicates that the HARMS_2_-AF score is a reliable tool for risk stratification, regardless of ethnic differences in AF pathophysiology and prevalence. Their study demonstrated that the HARMS_2_-AF score retains its predictive accuracy even in an Asian cohort, suggesting its broad applicability across different ethnic groups.

Unlike conventional risk models focused primarily on thromboembolic complications, the HARMS_2_-AF score incorporates modifiable lifestyle factors—hypertension, obesity, sleep apnea, smoking, and alcohol consumption—that are increasingly recognized as critical contributors to AF development and recurrence [10]. The HARMS_2_-AF score has the potential to shift clinical management by focusing on modifiable risk factors that can be managed through lifestyle interventions. This finding is consistent with recent literature that emphasizes the role of modifiable risk factors in AF management [11, 12]. Unlike many existing AF risk scores that focus primarily on non-modifiable factors such as age and genetics, the HARMS_2_-AF score incorporates modifiable lifestyle-related risk factors—including hypertension, elevated BMI, male sex, sleep apnea, smoking, and alcohol consumption—thereby emphasizing the potential for prevention through targeted lifestyle interventions. Strategies targeting weight management, blood pressure control, and reduction in alcohol consumption and smoking may substantially lower the risk of Ata recurrence. The identification of patients at higher risk for recurrence through validated scoring systems like HARMS_2_-AF can guide clinicians in making informed decisions regarding treatment options [11], including the need for intensified monitoring or alternative therapeutic strategies. Unlike more complex models that require extensive diagnostic tests, the HARMS_2_-AF score relies on easily obtainable clinical parameters, making it a valuable asset for routine clinical practice, especially in regions with limited access to advanced healthcare infrastructure. While our study did not collect detailed longitudinal data on lifestyle changes during the follow-up period, we recognize that modifications in lifestyle factors such as weight, alcohol consumption, and physical activity could influence the HARMS_2_-AF score over time. Future prospective studies should examine how changes in lifestyle risk factors throughout the post-ablation period impact Ata recurrence. This would allow for a better understanding of how ongoing lifestyle modifications could potentially reduce recurrence rates and improve long-term outcomes for AF patients. If further validated, the HARMS_2_-AF score has the potential to significantly alter clinical management of AF patients. By providing a more accurate prediction of Ata recurrence, the HARMS_2_-AF score can help clinicians identify high-risk patients who may benefit from more intensive monitoring and targeted interventions. This approach could lead to improved patient outcomes by reducing the incidence of Ata recurrence and associated complications. Moreover, the HARMS_2_-AF score could serve as an educational tool for patients, helping them understand their individual risk factors and the importance of adhering to lifestyle changes. This could enhance patient engagement in their own care, leading to better long-term adherence to treatment plans and lifestyle modifications.

Our study primarily focused on the role of the HARMS_2_-AF score in predicting Ata recurrence following RFCA. However, the electrophysiological characteristics observed during follow-up procedures and both the distribution of antiarrhythmic therapy play important roles in recurrence risk [13]. Among patients who experienced Ata recurrence, a substantial proportion (27.3%) underwent repeat ablation procedures. These procedures identified several key electrophysiological characteristics that may contribute to recurrence, including recovery of PV potentials, atrial tachycardia dependent on the mitral isthmus, left atrial roof tachycardia, and superior vena cava-originating AF. These findings highlight the importance of achieving complete PVI during the initial procedure and the potential need for targeting other arrhythmogenic foci in cases of recurrence. Overall, the presence of residual arrhythmogenic activity after the first ablation appears to be a key factor in Ata recurrence and emphasizes the role of repeat ablation in improving long-term outcomes. In addition, our analysis did not find a significant correlation between specific antiarrhythmic medications and Ata recurrence. This lack of correlation may be due to the relatively small sample size and retrospective nature of the study. Future studies should explore the role of antiarrhythmic therapy in more detail, including the timing and duration of therapy, to better understand its impact on recurrence rates.

The incidence and recurrence of AF exhibit differences between men and women, which are influenced by multiple factors. Men are more prone to AF due to several reasons. Physiologically, their higher androgen levels can affect cardiac electrophysiological activities by altering ion channel functions in cardiomyocytes, increasing the risk of AF [14]. Genetically, Y-linked genes/variants contribute indirectly to the higher risk of AF in males, although relevant research is still limited [15]. Also, men’s more common unhealthy lifestyles, such as smoking and excessive alcohol consumption, damage the cardiovascular system and contribute to the higher incidence of AF [16]. Previous studies have found that female AF patients have a higher recurrence rate after ablation [17, 18]. Physiologically, the decline in estrogen levels after menopause can lead to unstable cardiac electrical activity and promote atrial remodeling. They experience more obvious symptoms, higher heart rates, and potentially longer - lasting AF episodes, along with differences in cardiac structure like lower left atrial voltage, indicating more severe atrial lesions. Moreover, in clinical practice, women receive RFCA less frequently and are referred later, often after multiple anti - arrhythmic drug failures, when the condition is more severe, increasing the recurrence risk after ablation [19]. The interplay of sex hormones, genetics, and atrial substrate remodelling all contribute to these differences. Perhaps with the improvement of patients’ awareness of seeking medical treatment and the favorable medical conditions in the local area, our research results found that there was no statistical difference between gender and recurrence of AF after RFCA.

This study has several limitations that warrant consideration. First, as a single-center, retrospective cohort study with a relatively small sample size, the findings may be subject to selection bias and may not be generalizable to broader populations. Second, the follow-up assessments were limited to three discrete time points within the first year post-ablation, potentially missing late or intermittent episodes of Ata recurrence. Moreover, the reliance on 24-hour Holter monitoring at scheduled intervals, although consistent with current clinical practice, may have failed to capture asymptomatic or short-lived arrhythmias. The absence of continuous or long-term ECG monitoring likely resulted in underestimation of the true recurrence rate. Additionally, all ablation procedures were performed using a uniform protocol and standardized energy settings. While this ensured procedural consistency, it may reduce the applicability of our findings to other ablation techniques or individualized energy titration strategies. Another limitation is the lack of longitudinal data on post-procedural lifestyle changes. As a retrospective study, we were unable to evaluate whether improvements in modifiable risk factors influenced subsequent HARMS_2_-AF scores or impacted recurrence rates. Furthermore, the relatively high proportion of additional linear lesions in our patient cohort may have affected arrhythmia recurrence, potentially introducing a confounding variable.

Future research should aim to validate the predictive utility of the HARMS_2_-AF score in larger, prospective, multicenter studies. Extended follow-up durations and the incorporation of continuous rhythm monitoring strategies, such as wearable ECG devices or implantable recorders, may provide a more accurate assessment of recurrence. In addition, further investigation into the comparative effectiveness of alternative ablation protocols and energy delivery methods is warranted. Randomized controlled trials are needed to determine whether risk-guided interventions based on the HARMS_2_-AF score—particularly those targeting modifiable lifestyle factors—can reduce the incidence of post-ablation recurrence and improve long-term outcomes.

In conclusion, our study highlights the importance of the HARMS_2_-AF score, LAD, and CHA_2_DS_2_-VASc score in predicting the recurrence of AF after RFCA. These findings could guide clinicians in identifying high-risk patients and implementing targeted interventions to improve post-ablation outcomes. Future prospective studies are warranted to validate our findings and explore the potential benefits of lifestyle interventions in reducing Ata recurrence post-ablation.

## Data Availability

The data that support the findings of this study are available from the Kunshan Hospital of Traditional Chinese Medicine. Data are, however, available from the authors upon reasonable request from the corresponding author Jianhua Fan (E-mail: fjhheart@126.com) and with permission of the Kunshan Hospital of Traditional Chinese Medicine.

## Abbreviations

AF: Atrial fibrillation
RFCA: Radiofrequency Catheter Ablation
Ata: Atrial Tachyarrhythmia
BMI: Body Mass Index
LAD: Left Atrial Diameter
PAF: Paroxysmal Atrial Fibrillation
PsAF: Persistent Atrial Fibrillation
ECG: Electrocardiogram
TEE: Transesophageal Echocardiography
ICE: Intracardiac Echocardiography
PVI: Pulmonary Vein Isolation
PV: Pulmonary Vein
SR: Sinus Rhythm
LVEDD: Left Ventricular End-diastolic Diameter
LVEF: Left Ventricular Ejection Fraction
ROC: Receiver Operating Characteristic
AUC: Area Under The Curve
OR: Odds Ratio
CI: Confidence Interval
FHS: Framingham Heart Study
SPSS: Statistical Package for the Social Sciences
